# A Refractive Index Sensor Based on a Metal-Insulator-Metal Waveguide-Coupled Ring Resonator

**DOI:** 10.3390/s151129183

**Published:** 2015-11-19

**Authors:** Shu-Bin Yan, Liang Luo, Chen-Yang Xue, Zhi-Dong Zhang

**Affiliations:** 1 Science and Technology on Electronic Test &Measurement Laboratory, North University of China, No.3 Xueyuan Road, Taiyuan 030051, China; E-Mails: shubin_yan@nuc.edu.cn (S.-B.Y.); luoliang6616@sina.cn (L.L.); xuechenyang@nuc.edu.cn (C.-Y.X.); 2 Key Laboratory of Instrumentation Science & Dynamic Measurement, Ministry of Education, North University of China, Taiyuan 030051, China, No.3 Xueyuan Road, Taiyuan 030051, China

**Keywords:** surface plasmon polaritons, metal-insulator-metal waveguide, Fano resonance, refractive index sensor, finite-difference time-domain method

## Abstract

A refractive index sensor composed of two straight metal-insulator-metal waveguides and a ring resonator is presented. One end of each straight waveguide is sealed and the other end acts as port. The transmission spectrum and magnetic field distribution of this sensor structure are simulated using finite-difference time-domain method (FDTD). The results show that an asymmetric line shape is observed in the transmission spectrum, and that the transmission spectrum shows a filter-like behavior. The quality factor and sensitivity are taken to characterize its sensing performance and filter properties. How structural parameters affect the sensing performance and filter properties is also studied.

## 1. Introduction

Surface plasmon polaritons (SPPs) are charge density waves, formed by the interaction between incident photons and free electrons in a metal surface, that travel along the metal–dielectric interface [1]. Since SPPs are exponentially damped in the direction perpendicular to the metal-dielectric interface, they are tightly limited to the vicinity of metal surfaces. This results in near-field optical resolution that overcomes the diffraction limit [2,3]. Hence, photonic devices based on SPPs have found extensive applications in super-resolution imaging [4], non-linear optics [5], subwavelength optical integration [6], biochemical sensors [7], and advanced, ultra-high-density photonic integrated circuits [8].

SPP waveguides, including insulator-metal-insulator (IMI) waveguides [9], metal-insulator-metal (MIM) waveguides [10] and combined waveguides [11], have been widely studied. Among these waveguides, MIM waveguides, in particular, exhibit low transmission loss and strong localized field confinement [12,13,14], and have led to the development of sub-wavelength photonic devices such as splitters [15], couplers [16,17], and filters [18,19,20]. These devices are basically composed of waveguides and resonators. The resonator parameters determine their transmission properties. The coupling between a ring resonator and straight waveguides can generate a Fano resonance. Because the Fano resonance is extremely sensitive to the change in refractive index, ultra-high-sensitivity refractive index sensors can be constructed.

Here, a MIM waveguide-coupled ring resonator structure is discussed. In this study, to realize the Fano resonance, we etched one chip repeatedly, rather than multiple chips with different parameters, to adjust the coupling between the straight waveguides and ring resonator. The filter properties and the refractive index sensitivity are simulated by finite-difference time-domain (FDTD) methods. In addition, the effects of structural parameters on the transmission spectra are investigated.

## 2. Device Structure and Computational Methods

**Figure 1:**
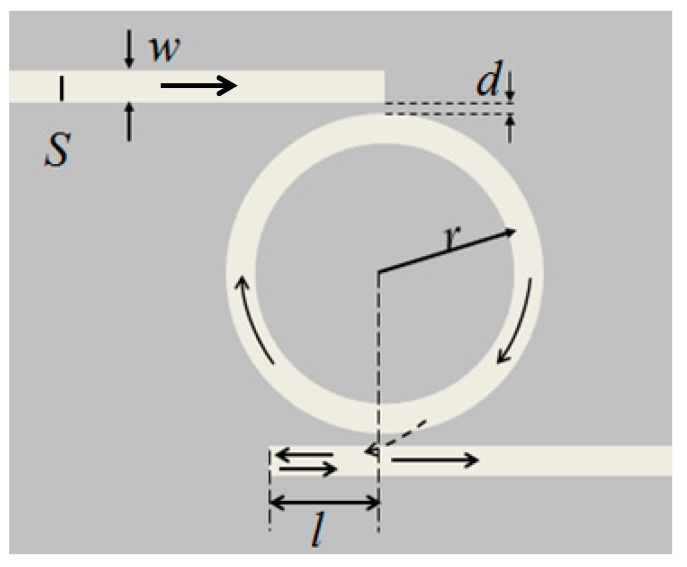
Schematic of the MIM waveguide-coupled ring resonator. *S* is the dipole source, *r* is the radius of the ring resonator, *d* is the coupling distance between the waveguide and ring, and *l* is the distance between the sealed end of the output waveguide and center of the ring resonator.

Figure 1 is a schematic of the MIM waveguide-coupled ring resonator, which consists of two straight MIM waveguides and a ring resonator. One end of each waveguide is sealed and the other end works as a port. In Figure 1, *S* is the dipole source, *r* is the radius of the ring resonator, *d* is the coupling distance between the waveguide and the ring resonator, and *l* is the distance between the sealed end of the output waveguide and the center of the ring resonator. To ensure that only the fundamental transverse magnetic mode is supported in the MIM waveguide, the widths *w* of the waveguides and ring resonator are fixed at 50 nm [21].

The frequency-dependent complex relative permittivity ε(ω) of silver is given by the modified Debye–Drude dispersion model [22]:

(1) $$\varepsilon \left(\omega \right)=\varepsilon_{\infty }+\left(\varepsilon_{s}-\varepsilon_{\infty }\right)/(1+i\omega \tau )+\sigma /i\omega \varepsilon_{0}$$

where the infinite permittivity *ε*_∞_ = 3.8344, the static permittivity *ε*_s_ = −9530.5, the relaxation time *τ* = 7.35 × 10^−15^ s, and the conductivity *σ* = 1.1486 × 10^7^ S/m.

The transverse magnetic mode equation for a MIM waveguide is [14,21]:

(2) $$tanh\;\left(\kappa w\right)=-2\kappa p\alpha_{c}/\left(\kappa^{2}+p^{2}{\alpha_{c}}^{2}\right)$$

where *κ* is wave vector in the MIM waveguide.

In Equation (2), *p* = *ε*_in_/*ε*_m_ and *α*_c_ = [*k*_0_^2^(*ε*_in_ − *ε*_m_) + *κ*]^1/2^, where *ε*_in_ and *ε*_m_ are the dielectric constants of the insulator and metal, respectively. The wave vector in free space is given by *k*_0_ = 2π/*λ*_0_, and *κ* can be determined with Equation (2). Thus, the real part of the effective index Re (*n_eff_*) of a MIM waveguide can be expressed as Re(*n_eff_*) = *ε_m_* + (*κ*/*κ*_0_)^1/2^. The effective wavelength *λ_spp_* of the SPPs can be obtained with the equation *λ_spp_ = λ*_0_/Re(*n*_eff_). Output energy flow *P_out_* is the integral of the *x*-component of the Poynting vector at the interface of the output port. The transmittance is given by *T* = *P*_out_/*P*_in_, where *P*_in_ is the input energy flow. We use the quality factor *Q* = *λ_0_*/*FWHM* to evaluate the filter properties of the MIM waveguide-coupled ring resonator, where *λ_0_* is the resonance wavelength and *FWHM* is the full width at half maximum of the resonance peak.

## 3. Results and Discussion

**Figure 2:**
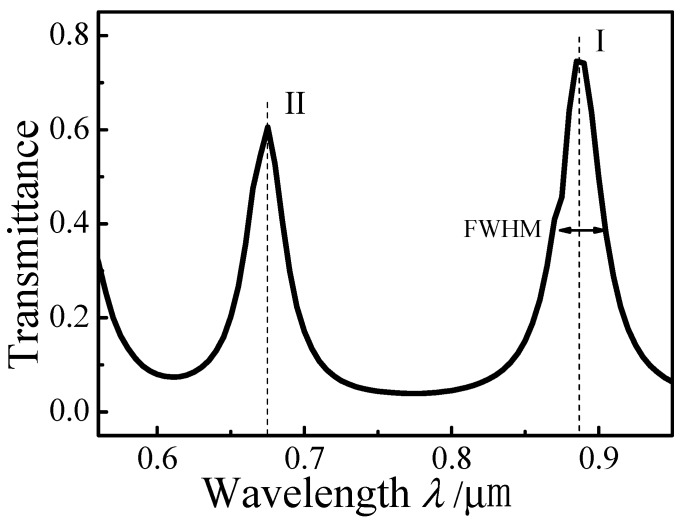
Transmission spectrum of the MIM waveguide-coupled ring resonator.

Figure 2 plots the simulated transmission spectrum of the MIM waveguide-coupled ring resonator with *d* = 6 nm, *r* = 300 nm, and *l* = 0 nm. There are resonances at peak I (*λ*_0_ = 0.887 μm) and peak II (*λ*_1_ = 0.675 μm). The *FWHM* of peak I is 0.034 μm, and that of peak II is 0.028 μm. Propagation of SPPs in the MIM waveguide-coupled ring resonator is shown in Figure 1. After SPPs are coupled into the ring, resonating SPPs are coupled into the output waveguide.

**Figure 3:**
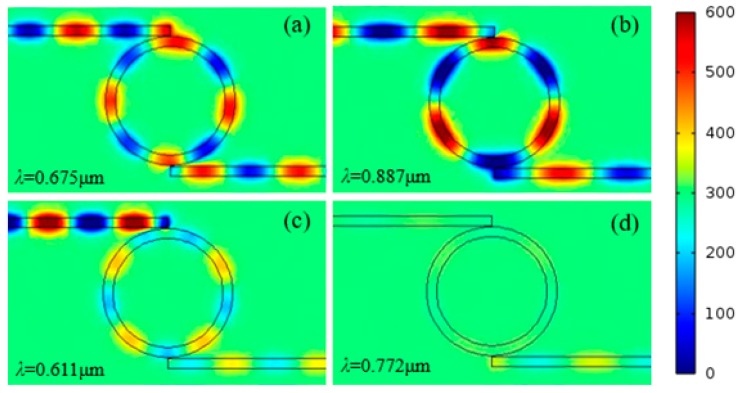
Steady-state distributions of the normalized magnetic field |*Hz*| at excitation wavelengths of (**a**) 0.675 µm; (**b**) 0.887 µm; (**c**) 0.611 µm; and (**d**) 0.772 µm.

To characterize the SPP resonances in the ring, the steady-state magnetic field |*Hz|* distributions are calculated. Figure 3a–d show the |*Hz*| field distributions of the MIM waveguide-coupled ring resonator structure at 0.675 µm, 0.887 µm, 0.611 µm, and 0.772 µm, respectively. Standing wave modes are formed in the ring. The |*Hz*| field distributions of the pass band correspond to the resonance peaks at 0.675 and 0.887 µm, respectively, while, the stop band distributions correspond to resonance peaks at 0.611 and 0.772 µm, respectively. In addition, standing waves help to explain the steady-state |*Hz|* distributions in the ring.

The standing wave condition is *L = m*(*λ*_spp_/2), where *m* is a positive integer. For *λ* = 0.675 µm, Re (*n_eff_*) of the MIM waveguide is 1.4367 and the corresponding effective SPP wavelength *λ_spp_* is 0.470 µm. Therefore, *m =* 2*L/λ_spp_* = 8, which agrees well with the numerical results in Figure 3a. For *λ* = 0.887 µm, Re (*n_eff_*) = 1.41, *λ_spp_* = 0.629 µm, and *m =* 6. For *λ* = 0.611 µm, Re(*n_eff_*) = 1.4529, *λ_spp_* = 0.421 µm, and *m* = 9. Finally, for *λ* = 0.772 µm, Re(*n_eff_*) = 1.4211, *λ_spp_* = 0.543 µm, and *m* = 7. Overall, the resonant wavelengths in Figure 2 basically follow *L = m* (*λ*_spp_/2). SPPs cannot be coupled into the output waveguide when *m* is an even number. The calculations based on the standing wave condition are consistent with the steady-state distributions of the normalized magnetic field |*Hz*| simulated with the FDTD method.

**Figure 4:**
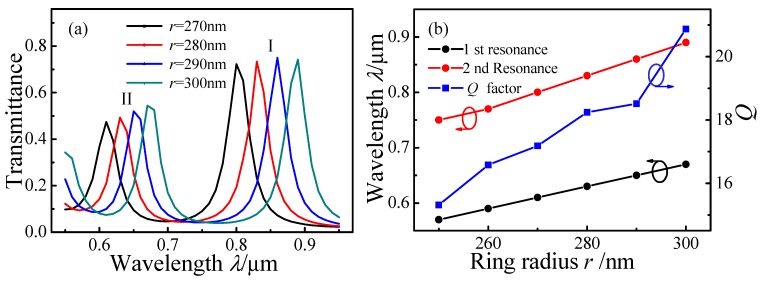
Simulated transmission spectra of MIM waveguide-coupled ring resonators as a function of radius *r*: (**a**) Spectra for *r* = 270 nm, 280 nm, 290 nm, and 300 nm; (**b**) Dependence of peak positions and *Q* factor on *r*.

To investigate how the radius *r* affects the transmission of the MIM waveguide-coupled ring resonator, it was increased from 270 nm to 300 nm in 10-nm increments with *d* = 6 nm and *l* = 0 nm. Figure 4a plots the simulated transmission spectra when *r* = 270 nm, 280 nm, 290 nm, and 300 nm. The transmittances of the I and II resonance peaks are approximately 0.7 and 0.5, respectively. With increasing *r*, the resonance peaks exhibit red shifts. When *r* > 290 nm, a new resonance peak emerges at shorter wavelength. Figure 4b plots the dependence of peak positions and *Q* factor on *r*. At increased *r*, both peaks exhibit red shifts and the *Q* factor increases. The coupling length between the straight waveguides and the ring resonator increases with increasing *r*, leading to a stronger coupling coefficient. In addition, the longer resonance length increases *Q* and the SPP filter has higher resolution at higher *Q*.

**Figure 5:**
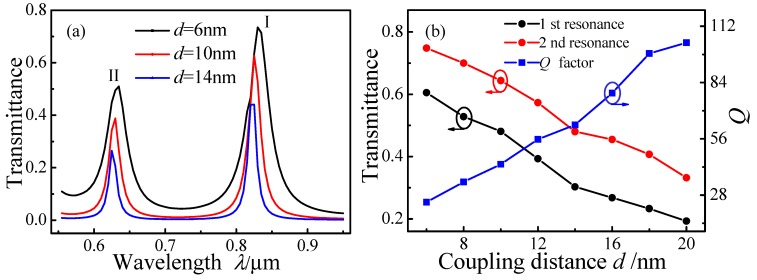
Transmission spectra of MIM waveguide-coupled ring resonators as a function of coupling distance *d*. (**a**) Spectra for *d* = 6 nm, 10 nm, and 14 nm; (**b**) Transmittance and *Q* factor as a function of *d.*

Figure 5 plots transmission spectra for MIM waveguide-coupled ring resonators as a function of *d* over the range 6–20 nm in 2 nm increments, with *r* = 300 nm and *l* = 0 nm. Both resonance peaks I and II exhibit slight blue shifts with increasing *d*, as shown in Figure 5a.

Figure 5b plots the transmittance of the two peaks and their *Q* factors as a function of *d*. The transmittance decreases with increasing *d*; however, the *Q* factor increases with increasing *d*. A smaller coupling coefficient (weaker coupling strength) will result in a decreased transmittance, a narrower *FWHM*, and a higher *Q* factor. Some of the SPPs that fail to form resonances in the ring dissipate because of ohmic heat loss. Accordingly, the noise will decrease, leading to an increased *Q* factor.

**Figure 6:**
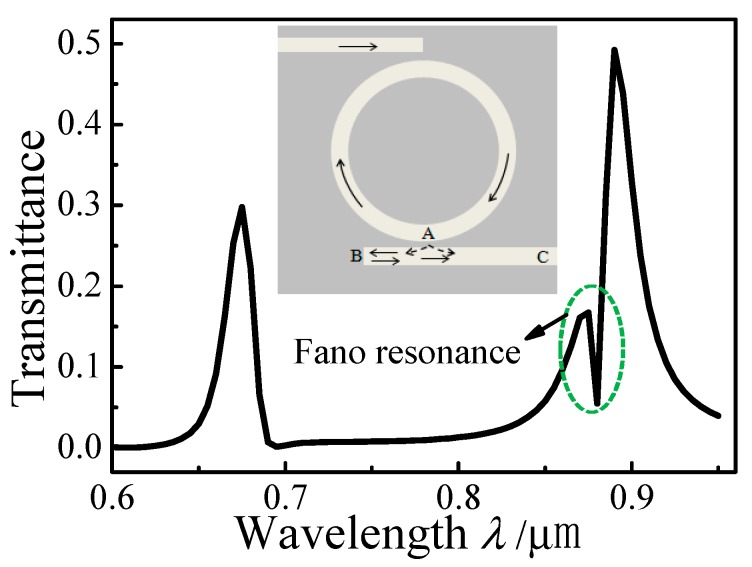
Transmission spectrum and SPP propagation path (inset) in a schematic of the MIM waveguide-coupled ring resonator.

Figure 6 plots the simulated transmission spectrum of the MIM waveguide-coupled ring resonator with *l* = 80 nm, and values of *w*, *r,* and *d* that are the same as those in Figure 2. A Fano resonance emerges on the left shoulder of resonance peak I. The inset in Figure 6 indicates the SPP propagation path. When the resonant SPPs in the ring resonator are coupled into the MIM waveguide, they split into two parts at position A. The SPP propagating towards C will form a broadband resonance, while the other SPP is reflected back in the sealed end (position B) and will form a narrowband resonance. As a result, a Fano resonance occurs from the destructive interference between the two SPPs. The transmittances of the resonance peaks in Figure 6 are smaller than those in Figure 2 because portions of the resonating SPPs are lost by the reflection at the sealed end of the output waveguide.

**Figure 7:**
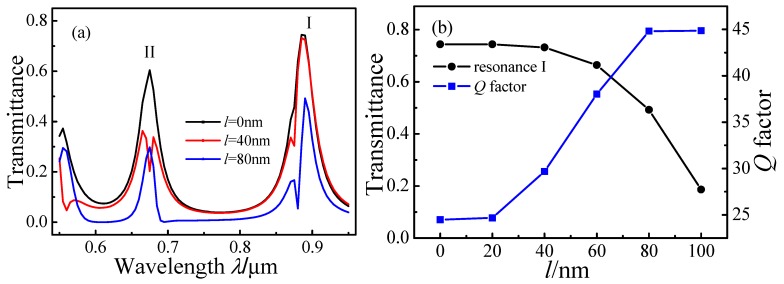
Transmission spectra of MIM waveguide-coupled ring resonators as a function of *l*. (**a**) Spectra for *l* = 0 nm, 40 nm, and 80 nm; (**b**) Transmittance of peak I and *Q* factor as a function of *l.*

Figure 7a plots the simulated transmission spectra for *l* = 0 nm, 40 nm, and 80 nm with fixed *r* = 300 nm and *d* = 10 nm. Resonance peak I splits into two resonance peaks when *l* ≥ 40 nm. The splitting increases with increasing *l*, revealing Fano resonances.

Figure 7b shows the transmittance and *Q* factor of resonance peak I at 0.887 µm as a function of *l*. The *Q* factor increases markedly as *l* increases from 20 nm to 80 nm. When *l* = 80 nm, the asymmetric line shape of peak I clearly reveals the Fano resonance and the smaller *FWHM* entails a larger *Q* factor.

**Figure 8:**
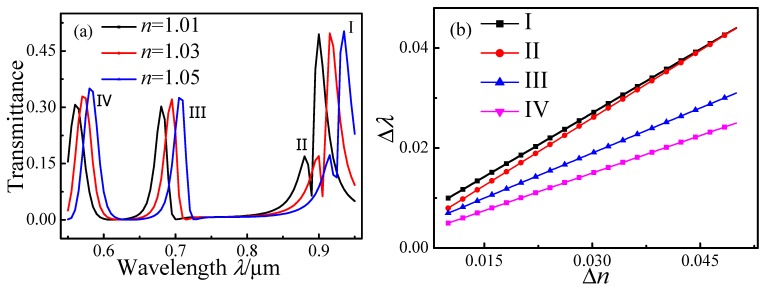
Transmission spectra of MIM waveguide-coupled ring resonators containing various dielectrics with refractive index *n*. (**a**) Spectra for structures containing dielectrics with *n* = 1.01, 1.03, and 1.05; (**b**) Peak red shifts as a function of Δ*n.*

To simulate the refractive index sensitivity of the Fano resonance, the MIM waveguide-coupled ring resonators were filled with dielectrics having various refractive indices *n*. The transmission spectra with *n* = 1.01, 1.03, and 1.05 for *l* = 80, *r* = 280 nm, and *d* = 10 nm are plotted in Figure 8a. Resonance peaks I–III are distinctly asymmetric, whereas peak IV has a Lorentzian line shape. All four peaks exhibit red shifts with increasing *n*. The value *δ* = Δ*λ*/Δ*n* quantitatively characterizes the refractive index sensitivity of the Fano and Lorentz peaks. As shown in Figure 8b, *δ* for the peaks I, II, III, and IV are 868.4, 921, 605.3, and 300, respectively. The Fano peaks are thus more sensitive to *n* variations than the Lorentzian peaks.

## 4. Conclusions

The transmission properties of a MIM waveguide-coupled ring resonator are simulated by FDTD methods. The transmission spectra display red shifts when the ring radius *r* increases. The transmittance decreases and the *Q* factor increases with increasing *d*, and the resonance peaks exhibit slight blue shifts. Fano resonance occurs when the sealed end of the output waveguide is moved from the ring center to the left. It is generated by the interference between the broadband resonance of the ring resonator and narrow-band resonance of the sealed end of the output waveguide. The Fano resonances exhibit a higher refractive-index sensitivity relative to that of the Lorentzian resonances. This study thus provides a pathway based on MIM waveguide-coupled resonators for designing sensors that detect refractive index changes. In addition, electromagnetic inverse scattering has revealed that neural networks can be used to verify ring resonator models because of their higher computational accuracy and optimization [23,24,25].
